# Structure, Mechanical Properties, and Rheological Characteristics of Poly(Butylene Adipate-co-Terephthalate)–Polylactic Acid Blends Modified via In Situ Maleic Anhydride Grafting

**DOI:** 10.3390/polym17162264

**Published:** 2025-08-21

**Authors:** Min Jin, Bei Qi, Kang Chen, Lijun Cao, Pengrui Chen, Ce Sun, Jianfeng Zhan, Zhuofeng Shao, Haiyan Tan, Yanhua Zhang

**Affiliations:** 1 State Key Laboratory of Woody Oil Resources Utilization, Northeast Forestry University, Harbin 150040, China; 2 Fujian Dazhuang Bamboo Industry Technology Co., Ltd., Jian’ou 353100, China; 3 College of Home and Art Design, Northeast Forestry University, Harbin 150040, China

**Keywords:** maleic anhydride, reactive blending, PLA, impact resistance

## Abstract

Polylactic acid (PLA) materials face inherent limitations in many applications due to their low toughness. To address this challenge, this study employed a reactive melt-grafting method to prepare maleic anhydride (MA)-grafted poly(butylene adipate-co-terephthalate) (PBAT–MA), providing an effective approach to improve the interfacial compatibility between PLA and PBAT, thereby significantly enhancing the toughness and impact resistance of PLA and expanding its application scope. The grafting reaction process of PBAT–MA was investigated, as well as its toughening mechanism and effect on PLA. The results showed that at a maleic anhydride concentration of 2 wt%, the obtained PLA–PBAT–MA composite material exhibited the best performance, with a fracture elongation of 358.1%, 450.4% higher than that of the unmodified composite material. The impact strength was 333.9 kJ/m^2^, 917.3% higher than that of the unmodified composite material. This enhanced effect is attributed to the optimal MA concentration preserving the tough structure of PBAT while effectively bridging the interface between PLA and PBAT, promoting efficient stress transfer between the two phases, and ultimately achieving exceptional toughness.

## 1. Introduction

With the rapid development of human civilization, white pollution caused by non-degradable plastics has gradually posed a threat to humanity itself [1,2]. In this context, it is imperative to explore the use of renewable plastics as alternatives to heavily polluting non-degradable plastics to better protect the environment [3,4]. Polylactic acid (PLA), a degradable eco-friendly material with high strength and excellent biocompatibility, has been extensively studied and applied [5,6]. It has been demonstrated to hold potential for substituting conventional petroleum-based polymers in industrial applications and other fields [7]. However, inherent drawbacks of PLA, such as poor impact resistance and low ductility, have significantly hindered its broader application [8,9]. Therefore, enhancing the toughness of PLA is critical to meet diverse application requirements [10]. Currently, primary methods to improve PLA performance include blending modification, modifying PLA chain structures, and incorporating small-molecule nucleating agents and plasticizers [11]. Blending PLA with flexible polymers such as polybutylene adipate terephthalate (PBAT) [12], polycaprolactone (PCL) [13], polybutylene succinate (PBS) [14], and polyethylene adipate (PEA) [15,16] represents the most direct and effective approach to enhance the mechanical properties of PLA-based materials.

Polybutylene adipate terephthalate (PBAT), a promising biodegradable plastic, exhibits outstanding toughness and serves as a viable alternative to non-biodegradable plastics [17]. However, its low modulus limits its applicability in many plastic-related applications. By blending PLA and PBAT to form composite materials, one can leverage their complementary advantages to improve PLA’s toughness, yet studies have shown that the mechanical performance of such composites remains suboptimal [18,19]. This is attributed to the high mixing entropy and poor compatibility between the aromatic structures in PBAT molecules and the aliphatic structures in PLA [20]. Consequently, the key to fabricating PLA–PBAT composites lies in achieving effective mixing of the two polymers, thereby compensating for their individual shortcomings and elevating overall performance [21,22]. The commonly employed method involves the addition of compatibilizers. The selection of compatibilizers is diverse. For instance, Sun et al. utilized alternative copolymers PSM-B, PSM-O, and PSM-D, synthesized from reactive maleic anhydride segments and imidazolium cations, as compatibilizers [23]. These effectively enhanced the elongation at break of PLA–PBAT composites and imparted excellent antibacterial properties. To effectively implement the grafting strategy, Nosova et al. proposed a method involving the use of a free radical mechanism to graft a polyperoxide layer onto the surface [24]. This approach ensured the uniform dispersion of free radicals across the material’s surface, thereby guaranteeing the controllability of the grafting process. A proven compatibilization strategy involves utilizing peroxide-activated anhydrides. Maleic anhydride (MA), widely employed as a compatibilizer in reactive blending processes involving polyesters, demonstrates high reactivity with hydroxyl groups, facilitating efficient polymer grafting [25]. The anhydride group in maleic anhydride exhibits high reactivity towards hydroxyl groups, and both PLA and PBAT contain terminal hydroxyl groups in their structures that can serve as reactive sites, enabling effective bonding. Additionally, MA’s low tendency to self-polymerize ensures the feasibility of grafting reactions [26]. This can ensure that it mainly connects to the molecular chain segments to create more reaction sites.

The underlying mechanism responsible for enhancing the compatibility between PBAT and PLA through the incorporation of MA is multifaceted. Specifically, under the catalysis of peroxides, PLA and PBAT generate corresponding free radicals, which subsequently react with maleic anhydride to promote the formation of graft copolymers, namely PLA–MA–PLA, PBAT–MA–PBAT, and PLA–MA–PBAT, as well as the development of highly branched or cross-linked structures [27]. In previous studies, MA has primarily been incorporated by grafting onto PLA to form PLA-g-MA, which is subsequently blended with PBAT or used in small quantities as a compatibilizer. For instance, Phetwarotai et al. synthesized PLA-g-MA through MA grafting and incorporated it as a compatibilizer into PLA–PBAT–PET composites, which significantly enhanced the mechanical properties of the composite material [28]. They attributed this improvement to the formation of ester bonds in PLA-g-MA, which contributed to superior mechanical performance. Teamsinsungvon et al. explored the impacts of grafting MA onto PLA on the mechanical, thermal, and morphological characteristics of the composite material. Their research results demonstrated that the degree of interfacial adhesion of the PLA–PBAT blend with grafted PLA was enhanced and the mechanical properties improved [29]. Nevertheless, the effects of existing methods for improving the toughness of the PLA–PBAT blend through grafting PLA have been found to be insignificant. In PLA–PBAT composites, PLA is generally regarded as the primary contributor to material strength, while PBAT provides the essential toughness. Previous studies have demonstrated that PLA-g-MA significantly enhances the tensile strength of the composite material. We hypothesize that during the reaction between MA and PLA, partial internal cross-linking may occur, forming a PLA–MA–PLA structure with higher structural integrity, but reduced toughness. Therefore, to improve the overall ductility of the composite, an alternative approach would be to modify PBAT with MA instead. Miao et al. investigated the effects of different amounts of PBAT–MA on PLA–PBAT composites using a commercially available PBAT–MA and a self-made PBAT–MA. The results demonstrated that an appropriate amount of PBAT–MA enhanced the compatibility of PLA–PBAT composites, thereby improving their toughness [30]. Similarly, Nam et al. prepared PLA-g-MAH, PBAT-g-MAH, and (PLA–PBAT)-g-MAH separately and studied their impact on PLA–PBAT composites. The findings revealed that the incorporation of maleic anhydride significantly improved the compatibility of PLA–PBAT composites, leading to a notable enhancement in their mechanical properties [31]. Strategically modifying PBAT by grafting MA can optimize the compatibility between PBAT and PLA, thereby promoting the formation of PLA–MA–PBAT structures and reducing the production of PLA–MA–PLA structures. This ultimately leads to an effective enhancement of the overall toughness of the blend. However, existing research still lacks sufficient structural characterization and mechanistic understanding of PBAT–MA.

In this research, MA was employed as a modifier, with benzoyl peroxide (BPO) serving as the initiator, to fabricate MA-modified PBAT. Subsequently, the modified PBAT was melt-blended with pure PLA, yielding the toughened PLA–PBAT blend. The success of grafting was affirmed by characterizing the molecular structure of the modified PBAT. To illuminate variations in the toughness of the blend and its intrinsic mechanism, a comparative analysis was conducted on the modified PLA–PBAT blend, its unmodified counterpart, and the unmodified blend. This comparative analysis encompassed a comprehensive study of mechanical properties, thermal behavior, microstructure morphology, and rheological properties. The objective of this research was to explore the reaction process of grafting MA onto PBAT, as well as its influence on the properties of PLA, and to analyze the mechanism of toughening PLA by grafted PBAT. It provides insights for expanding the scope of application of PLA.

## 2. Experimental

### 2.1. Materials

PLA (FY602, 1.24 g/m^3^, melting temperature of 160 °C) in the form of colorless transparent granules was provided by Anhui Fengyuan Group Co., Beijing, China. Its average molecular weight is 140,000 g/mol and the melt index is 9 g/10 min (190 °C/2.16 kg). PBAT (TH801, 1.25 g/m3, melting temperature of 110 °C) in the form of white granules was provided by Lan Shan Tunhe Chemical Industry Co., Shanghai, China) Benzoyl peroxide (C_14_H_10_O_4_, A.R., 98%, Sinopharm Chemical Reagent Co., Shanghai, China). MA (AR, 99.5%, MW = 98.06) was purchased from McLin Company.

### 2.2. Synthesis of PBAT-Grafted MA (PBAT–MA)

Firstly, PBAT was dried at 70 °C for 12 h. Subsequently, MA-grafted PBAT was prepared in a twin-screw extruder (SHJ-20, Nanjing Jiente Machine Point Co., Nanjing, China.) by blending PBAT, MA, and benzoyl peroxide. During the processing, a twin-screw extruder with a screw ratio of 40 and rotating in the same direction was used. The feeding rate was set to 12 rpm, and the screw speed was maintained at 60 rpm. The temperatures of the five zones were set to 125/130/135/135/130 °C. The MA content was 1 wt%, 2 wt%, 3 wt%, and 5 wt%, respectively, and the amount of BPO was fixed at 1 wt%. All PBAT–MA processed from the above conditions was crushed by a crusher and then used later.

### 2.3. Preparation of Blends

Firstly, PLA, PBAT, and PBAT–MA were dried at 70 °C for 12 h. Subsequently, they were blended by means of a twin-screw extruder. The ratio of PLA to PBAT or PBAT–MA was fixed at 70:30. During the processing, except for the feeding rate being increased to 36 rpm, all other parameters remain as mentioned above. The temperatures of the five zones were set to 175/175/180/175/175 °C. Table 1 presents the composition of the composite materials and their corresponding numbers. Next, the fabricated samples were crushed using a crusher, and ultimately test specimens were fabricated using dumbbell-shaped and rectangular molds in an injection molding machine (WZS10D, Shanghai Xinshuo Precision Machinery Co., Shanghai, China) under conditions of 180 °C and 0.6 MPa.

### 2.4. Characterization and Material Performance Testing

#### 2.4.1. Fourier Transform Infrared Spectroscopy (FTIR)

The samples were scanned by employing a Tensor II type Fourier transform infrared spectrometer (Bruker, Hong Kong). The scanning range of the samples was set within 4000–400 cm^−1^ with a scanning precision of 4 cm^−1^. The thickness of all samples was controlled within the range of 0.2–0.3 mm. All samples were scanned 32 times.

#### 2.4.2. Mechanical Properties

The mechanical properties of the samples were evaluated using a CMT-5504 electronic universal mechanical testing machine (SANS, Shenzhen, China) and a 5000 N sensor (BSS-500 kg, transcell, United States). Tensile tests were performed according to the GB/T 1040.1-2018/ISO-527-1 standards using dumbbell-shaped specimens with dimensions of 75 × 5 × 2 mm^3^, a narrow cross-section width of 5 mm, and a test speed of 5 mm/min [32]. The test splines were all 1BA-type splines that were injection-molded and met the ISO-527-2 standard [33]. The unnotched impact toughness of the composite was assessed using a Charpy impact tester (JC-25, Chengde Precision Testing Machine Co., Chengde, China), following ISO-179 [34]. The impact-test specimens were molded from molds with dimensions of 80 × 10 × 4 mm^3^. Prior to the tensile and impact tests, the dimensions of each specimen were measured using a vernier caliper. At least five tests were conducted for each composition, and the results were averaged. All tests were performed at ambient temperature.

#### 2.4.3. Differential Scanning Calorimetry

Differential scanning calorimetry (DSC 214 Polyma, NETZSCH Instruments, Selb, Germany) was employed for thermal analysis of PLA, PBAT, and PLA–PBAT blends. Samples were heated from 25 °C to 210 °C at a rate of 10 °C/min under a nitrogen atmosphere, followed by an isothermal hold for 5 min to eliminate thermal history. Subsequently, the samples were cooled to 25 °C at a rate of 10 °C/min and reheated to 210 °C at 5 °C/min for the second heating scan, from which the thermal behavior of all blends and composites was analyzed. The crystallinity ($x_{c}$) of PLA was estimated using Equation (1):

(1) $$x_{c}\left(\%\right)=\frac{∆H_{m}}{\left(0.7∆H_{m,PLA}+0.3∆H_{m,PBAT}\right)}$$

where $∆H_{m}$ is the specific melting enthalpy of the blend from the second heating trace, and $∆H_{m,PLA}$ and $∆H_{m,PBAT}$ are the specific melting enthalpies of 100% crystalline PLA (93.6 J/g) and PBAT (114.0 J/g) [35], respectively.

#### 2.4.4. Thermogravimetric Analysis

Thermogravimetric analysis (TGA) was performed using a thermogravimetric analyzer (209 F3, NETZSCH Instruments, Selb, Germany) under a nitrogen atmosphere (100 mL/min). Samples of 5 mg were heated from 30 °C to 600 °C at a rate of 10 °C/min, and the weight loss curve and weight loss rate curve were recorded.

#### 2.4.5. Dynamic Mechanical Analysis

Dynamic mechanical analysis (DMA) was performed using a dynamic mechanical analyzer (242 E, NETZSCH, Selb, Germany) in three-point bending mode. Dumbbell-shaped composite specimens with dimensions of 45 mm × 5 mm × 2 mm were tested at a heating rate of 5 °C/min under 1 Hz frequency, with the temperature program spanning from −20 °C to 150 °C.

#### 2.4.6. X-Ray Diffraction (XRD)

The wide-angle X-ray diffraction of the samples was tested employing a Rigaku Corporation (Japan, D/max 220) X-ray diffractometer, with a scanning speed of 2°/min over the range of 2θ = 5–55°.

#### 2.4.7. Scanning Electron Microscopy (SEM)

The surface morphologies of the brittle fracture sections of PLA and composite materials were observed by a Quanta 220 scanning electron microscope (FEI Company, Hillsboro, OR, USA) at a voltage of 10 kV. All the samples were fabricated by subjecting injection-molded dumbbell-shaped specimens to liquid nitrogen immersion and subsequent brittle fracture.

#### 2.4.8. Rheological Properties

The rheological behavior of the blend was analyzed using a rotational disk rheometer (TA Instruments, New Castle, DE, USA) with the AR2000EX model. Thickness-wise, the circular samples were cut to 1 mm, and diameter-wise, they were 25 mm. The measurements were conducted at a frequency range of 0.628 rad/s to 628 rad/s at a controlled strain of 0.1% under an air atmosphere. Among the tested samples, pure PBAT and PBAT–MA were analyzed at 140 °C, while pure PLA, pure PBAT, and the remaining composite materials were measured at 180 °C. Notably, pure PBAT underwent two independent measurements to ensure compliance with the corresponding conditions.

#### 2.4.9. Dynamic Mechanical Thermal Analysis

Dynamic mechanical thermal analysis (DMTA) was conducted using the three-point bending mode of a DMA 242 E (NETZSCH, Selb, Germany) dynamic mechanical analyzer to study the dynamic thermal mechanical properties of PLA, PBAT and their blends. The test temperature gradually increased from −20 °C to 150 °C, with a heating rate of 5 °C per minute and a frequency of 1 Hz.

#### 2.4.10. Gel Permeation Chromatography

The molecular weights of pure PBAT and synthesized PBAT–MA samples were determined using an ultraperformance liquid chromatography (1260 Infinity II, Agilent, Santa Clara, CA, USA). For each sample, 1–3 mg was dissolved in 1 mL of tetrahydrofuran (THF) prior to analysis.

#### 2.4.11. Nuclear Magnetic Resonance Spectroscopy

The chemical structure of the samples was characterized using nuclear magnetic resonance (NMR) spectroscopy. Each sample, calibrated with deuterated chloroform (CDCl_3_), was scanned at room temperature under a Bruker Avance III HD 500 MHz spectrometer (Bremen, Germany) to obtain the corresponding ^1^H NMR spectrum.

## 3. Results and Discussion

### 3.1. Chemical Modification of PBAT

**Figure 1:**
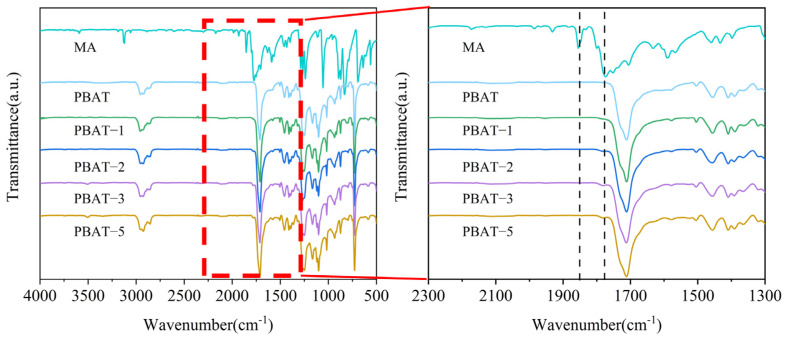
Infrared spectra of MA, PBAT, and PBAT–MA.

The FTIR spectra of MA, PBAT, and MA-modified PBAT with different MA content are shown in Figure 1. Pure MA exhibits characteristic peaks at 1775 cm^−1^ and 1855 cm^−1^, corresponding to the symmetric and asymmetric stretching vibrations of cyclic carbonyl groups [36]. PBAT shows a characteristic peak at 1711 cm^−1^, representing carbonyl groups attached to the benzene ring. After MA modification, a new peak appears at 1775 cm^−1^ in the spectrum of MA–PBAT that is absent in pure PBAT, indicating successful grafting of MA onto the PBAT.

**Figure 2:**
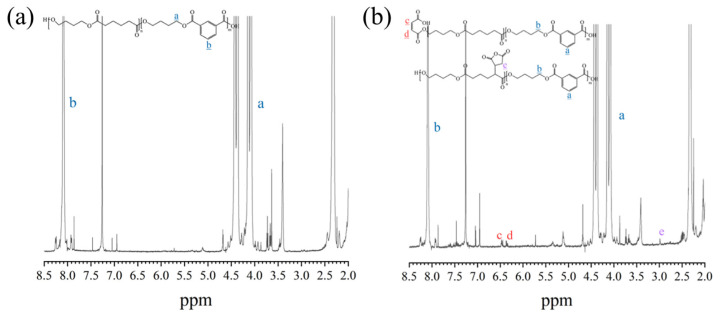
^1^H-NMR spectra of (**a**) pure PBAT and (**b**) PBAT–MA. The letters in the figure represent the positions of hydrogen atoms and the corresponding peaks.

**Figure 3:**
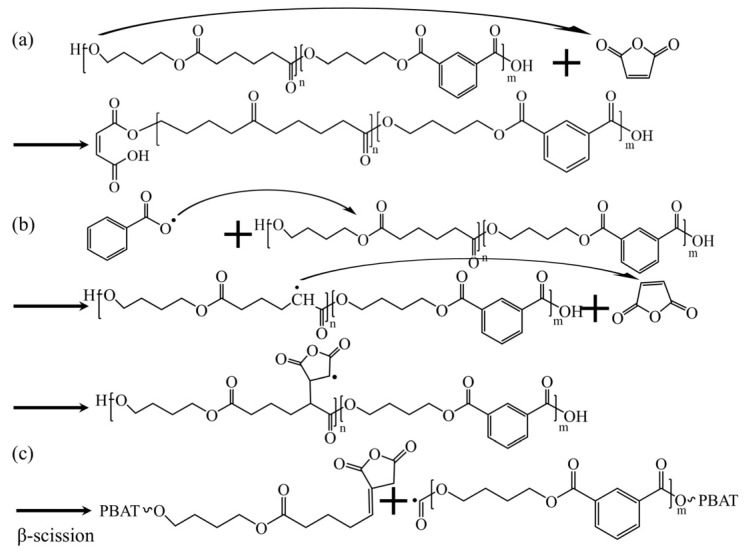
(**a**,**b**) Two predictions of the reaction mechanism between PBAT and MA, and (**c**) possible chain-breaking reactions.

Figure 2a,b present the ^1^H NMR spectra of PBAT and PBAT–MA. Figure 3 presents (a) and (b) two proposed reaction mechanisms between PBAT and MA and (c) a potential chain-scission reaction. Pure PBAT exhibits significant ^1^H-NMR resonance signals at 4.5–4.5 ppm and 8.2 ppm, which mainly originate from the methylene protons (a) of the long chain of PBAT and the secondary methyl protons (b) of the benzene ring. Compared with the spectrum of pure PBAT, the spectrum of PBAT–MA shows new resonance signals at 6.4 ppm and 3.0 ppm nearby (c), (d), and (e). The two new peaks at 6.4 ppm nearby correspond to the vibrations of the secondary methyl protons of the carbon–carbon double bond in the MA used in Figure 3 reaction (a), as the two hydrogen atoms are in different chemical environments, resulting in two equal, but different peak values. The new peak at 3.0 ppm corresponds to the vibrations of the hydrogen atoms in the succinic anhydride used in Figure 3 reaction (b) during grafting. The appearance of these three vibration peaks provides strong evidence that MA has been successfully grafted onto the polymer through the above two reactions.

**Figure 4:**
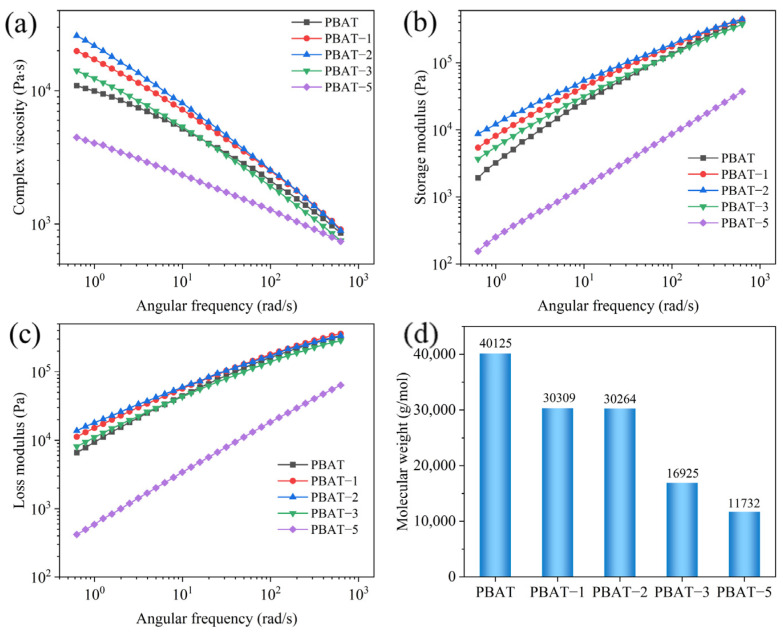
(**a**) Complex viscosity, (**b**) storage modulus, (**c**) loss modulus, and (**d**) number-average molecular weight of PBAT and PBAT–MA.

The rheological test results of PBAT–MA modified with different concentrations of MA are shown in Figure 4. Figure 4a–c present the curves of complex viscosity, storage modulus, and loss modulus of the samples as functions of angular frequency, respectively. It can be observed that all samples exhibit varying degrees of shear-thinning behavior with increasing shear rate. As the MA concentration increases, the viscosity, storage modulus, and loss modulus of the samples initially rise and then decline, reaching their maximum values at a concentration of 2 wt%. This is attributed to the grafting reaction, which effectively enhances intermolecular interactions, restricting chain mobility and thereby improving rheological properties. However, upon further increasing the MA concentration, all parameters decrease rapidly, falling below those of pure PBAT at a concentration of 5 wt%. Based on the two proposed reaction pathways depicted in Figure 3, it can be inferred that under high MA concentration conditions, the reaction illustrated in Figure 3b predominates. This occurs because reaction (b) further triggers reaction (c), wherein radicals undergo beta-scission within the molecular chain segments. This results in shortened chain segments and reduced resistance to chain movement, thereby decreasing viscosity, storage modulus, and loss modulus.

To further validate this hypothesis, the molecular weight of PBAT–MA was measured via GPC, with the results presented in Figure 4d. As shown, pure PBAT exhibits the highest molecular weight, while PBAT–MA demonstrates an initial slight decrease followed by a rapid decline in molecular weight. At 2 wt% MA concentration, the molecular weight remains comparable to that at 1 wt%, indicating that the reaction between MA and PBAT primarily follows the pathways illustrated in Figure 3a,b. However, with further increases in maleic anhydride concentration, under high MA loading, the radical-induced reaction promotes chain scission in PBAT, shifting the dominant pathway toward the mechanism depicted in Figure 3c. Macroscopically, this manifests as a reduction in molecular weight, which aligns with the earlier hypothesis.

### 3.2. Mechanical Property Analysis

**Figure 5:**
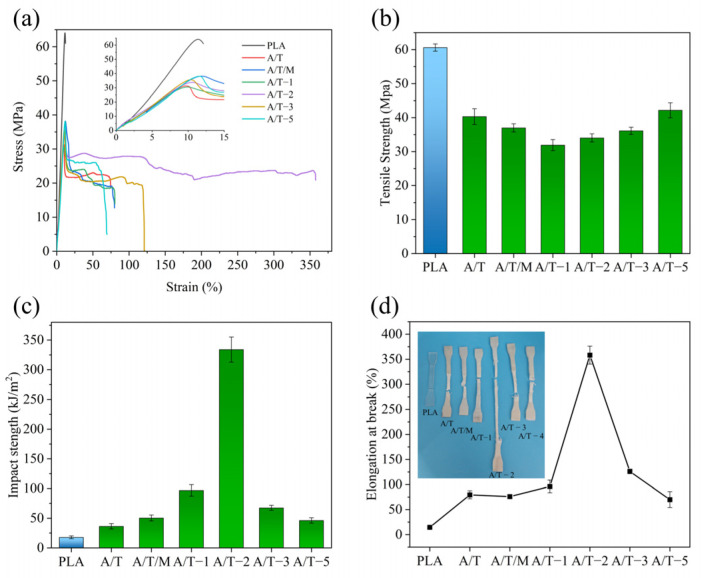
Mechanical property test results of composites: (**a**) stress–strain curves, (**b**) tensile strength, (**c**) unnotched impact strength, and (**d**) elongation at break of PLA–PBAT blends with different ratios of MA.

The mechanical properties of PLA composites are summarized in Table 2 and depicted in Figure 5. It can be observed that the elongation at break of the unmodified PBAT in the A/T sample is merely 79.5%. When PBAT is modified using MA, MA–PBAT significantly enhances the toughness of the A/T composites. The tensile elongation of A/T-2 group with MA addition of 2 wt% reaches its maximum value of 358.1%, which is four to five times that of the initial material. Nevertheless, it can also be noted that the strength of the material drops from 40.3 MPa to 34.0 MPa, a decrease of 15.6%. The strength of A/T-5 gradually ascended to 42.2 MPa under the influence of more MA, yet the elongation at break declined to 69.9%. By comparing the mechanical properties of the two materials (A/T-2 and A/T/M, both containing 2 wt% MA), it is evident that pre-modification followed by blending can conspicuously enhance the ductility of materials. This indicates that the MA grafted onto the modified PBAT provides more sites for binding with PLA, reducing the formation of PLA–MA–PLA that might occur when MA is directly added, thereby enabling more force from PLA to be transmitted to PBAT during stress and slowing the fracture. However, when the MA concentration reaches 5 wt%, the cross-linking reaction of MA causes extensive breakage of PBAT chain segments, thereby disrupting the tough structure and leading to a sharp decline in elongation at break. At this stage, however, the presence of numerous small PBAT–MA fragments with abundant reactive sites enables enhanced bonding with PLA, consequently improving the tensile strength.

The impact properties of the blends are presented in Table 2. PLA has always been limited in its application due to its relatively low impact strength. Therefore, improving its impact properties is also a key aspect in enhancing its toughness. With the increase in the content of MA, the impact strength of the blends is enhanced. The impact strength of the A/T composite without MA reaches 36.4 kJ/m^2^, which is twice that of pure PLA. The impact strength of the A/T composite with a 2 wt% concentration of MA can reach 333.9 kJ/m^2^, which is 19.2 times that of the sample without addition. The reason can be considered to be the PLA–MA–PBAT copolymer formed by the PBAT grafted with MA and PLA. PLA–MA–PBAT connects the two interfaces, improving the compatibility. The impact stress on PLA is well transferred to the PBAT molecular chains through MA, thereby requiring more energy for the composite material to be damaged.

### 3.3. Surface Micromorphology Analysis

**Figure 6:**
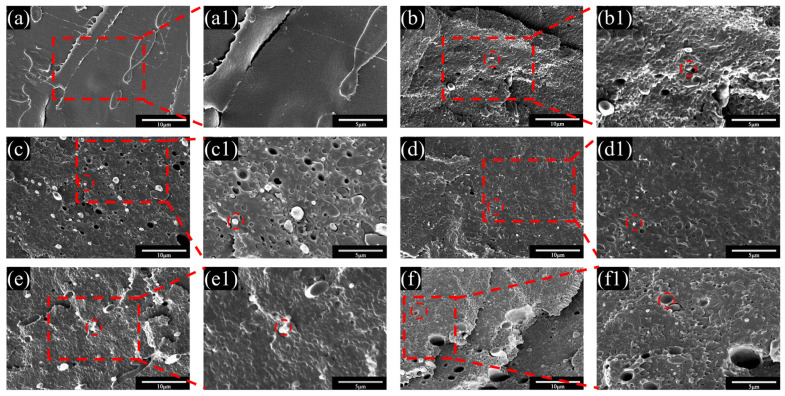
SEM images of fractured surfaces at 5000× magnification for (**a**) pure PLA, (**b**) A/T, (**c**) A/T-1, (**d**) A/T-2, (**e**) A/T-3, and (**f**) A/T-5 and at 10,000× magnification for (**a1**) pure PLA, (**b1**) A/T, (**c1**) A/T-1, (**d1**) A/T-2, (**e1**) A/T-3, and (**f1**) A/T-5.

The compatibility of composite materials was characterized through microscopic morphology. Figure 6 presents the SEM images of the cryogenically fractured cross sections of PLA blends. It can be observed that in Figure 6a, the surface of pure PLA is smooth, while in Figure 6b, the A/T exhibits a distinct island structure, with PBAT embedded in the PLA matrix in the form of larger particles, suggesting poor compatibility between the two. After the modification of PBAT with MA, it can be seen that the pores in the PLA and the particle diameter of PBAT have decreased, and the interface between the two phases has gradually become blurred. The reduction in the sea–island structure indicates effective stress transfer better between the two phases. Nevertheless, with the increase in the amount of MA added, the degree of cross-linking among PBAT molecules has deepened. In Figure 6e of the A/T-3 blend and Figure 6f of the A/T-5 blend, larger and more PBAT particles are presented, which has significantly affected their toughness, but slightly enhanced a portion of their strength. On a comprehensive comparison basis, the A/T-2 blend in Figure 6d shows a more uniform distribution and a closer interface between the two phases, which echoes and explains part of the reason for its superior mechanical properties.

### 3.4. Thermal Properties and X-Ray Diffraction Analysis

**Figure 7:**
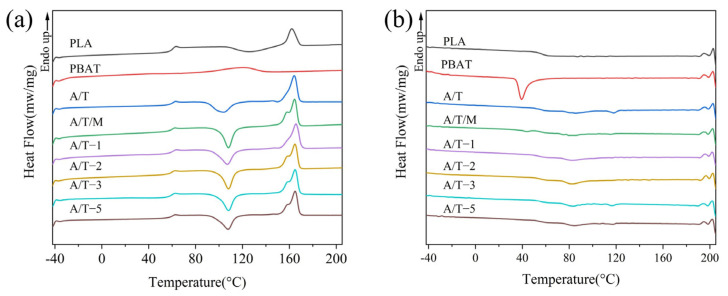
DSC curves of PLA, PBAT, and their blends for (**a**) the second heating and (**b**) the first cooling processes.

Figure 7 shows the DSC curve of the mixture, while the corresponding thermodynamic property data are given in Table 3. It can be observed that compared with PLA, the T_cc_ of the A/T blend decreases by approximately 20 °C, a phenomenon that has also been noted in other studies [37]. This suggests that the addition of PBAT enhances the crystallization performance of PLA in the blend, increasing the crystallinity from 0.8% in pure PLA to 3.2%. Nevertheless, the incorporation of MA leads to a rebound in T_cc_, once again suppressing the crystallization ability. However, after treatment with 2 wt% MA, it can remain at 4.3%. Furthermore, the T_g_ of pure PLA and pure PBAT are 60.2 °C and −30.1 °C. With the increase in MA, the difference in T_g_ in the samples manifested a tendency of first decreasing and then increasing, reflecting the variations in compatibility. However, as the concentration of MA increases, the difference once again widens, which might be attributed to excessive cross-linking. When the MA content is 2 wt%, the composite material has the lowest ΔT_g_ of approximately 90.1 °C, indicating the best compatibilization effect, which is in line with the previous observations. From the cooling curves in Figure 7b, it can be observed that pure PBAT exhibits a distinct crystallization peak within the temperature range of 30–60 °C, indicating its relatively high crystallinity. In contrast, neither pure PLA nor the composite materials show clear crystallization peaks, which is consistent with their lower crystallinity observed in the heating curves. Nevertheless, the composites display a broad exothermic peak of 45–120 °C, suggesting that PBAT contributes to enhanced polymer crystallization to some extent.

**Figure 8:**
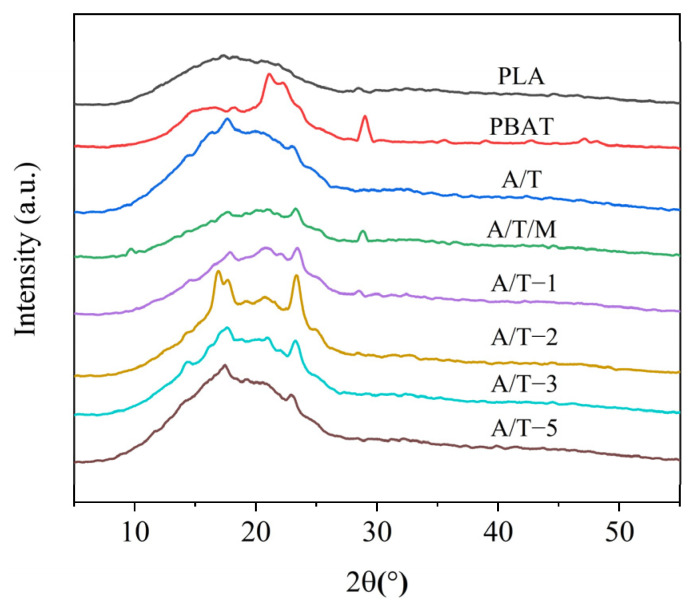
XRD curves of PLA, PBAT, and their blends.

The XRD patterns of samples are presented in Figure 8. Pure PLA exhibits no obvious diffraction peaks, and thus it can be regarded as amorphous. PBAT, however, demonstrates four diffraction peaks at 16.4°, 18.1°, 21.1°, and 23.3°, indicating the existence of a crystalline structure in PBAT, which is in accordance with the DSC data results. It can be observed from the XRD patterns of the A/T blends that the diffraction peaks of the curves are generally consistent with those of PBAT. Hence, it is believed that the crystalline portion in the blends is the crystallization of PBAT. It can be further discerned from the figure that after the addition of MA, the 16.4° diffraction peak undergoes a process of decreasing, increasing, and then decreasing again. The enhancement of crystallinity in DSC might be related to this, and it can be assumed that the molecular chain segments of PLA and PBAT are closer.

**Figure 9:**
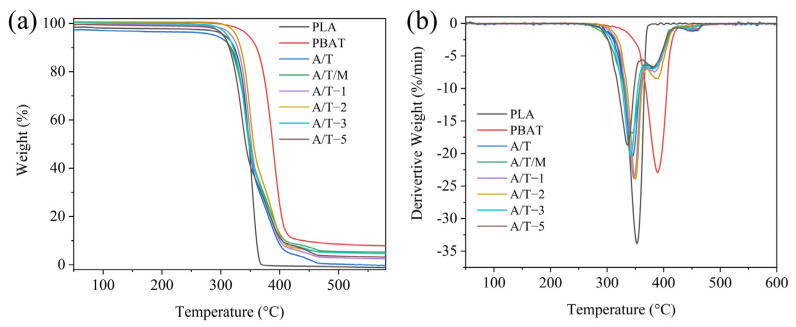
(**a**) TGA and (**b**) DTG curves of PLA, PBAT, and their blends.

The TG and DTG curves of PLA blends are depicted in Figure 9. The corresponding initial thermal degradation temperatures and the temperatures at 50% and 95% (final) mass loss of these materials are listed in Table 4. It is not difficult to observe that the pure polymer undergoes a one-step thermal degradation process, while the other composite materials exhibit distinct two thermal degradation stages, corresponding to the decomposition processes of the two phases. PLA commences decomposition at 311.8 °C and terminates at 369.4 °C, with essentially no residue remaining after the process. PBAT, conversely, initiates decomposition at 344.7 °C and concludes at 386.3 °C, generating residues. This indicates that PBAT possesses superior thermal stability compared to PLA, which has also been addressed in other literature [29]. In contrast to the A/T materials, the materials with MA content of 1 wt% and 2 wt% demonstrate higher thermal stability. Among them, the initial decomposition temperature and final degradation temperature of the material with a 2 wt% MA content are approximately 14 °C higher than those of the material without MA. This might be attributed to the formation of stronger chemical bonds between PBAT and PLA treated with MA, thereby enhancing the binding energy. Regarding the group where maleic anhydride was added directly during blending, the thermal stability of the resulting product was poorer. It is hypothesized that after the addition of maleic anhydride, it reacts simultaneously with PLA and PBAT, forming incompatible molecular chain segments (PLA-g-PLA or PBAT-g-PBAT). This will exacerbate the ester exchange reaction during thermal decomposition, thereby reducing the thermal stability. Likewise, this reasoning is applicable to cases with a higher additional amount of MA. The higher the concentration of MA, the more prone it is to occupy the reaction sites on the PBAT chain segments and cross-link with each other. However, this cross-linking occurs during the formation of PBAT–MA, leading to the scission of molecular segments within the PBAT chains. During thermal degradation, the cleavage of molecular segments constitutes one of the mechanisms of thermal degradation. Consequently, the already reduced molecular weight further accelerates the thermal decomposition of the resulting composite material, thereby compromising its thermal stability.

**Figure 10:**
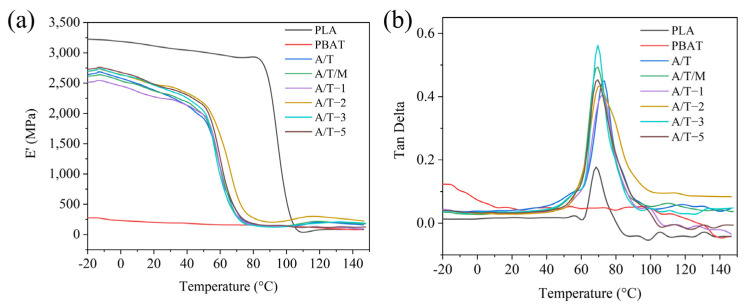
(**a**) Storage modulus and (**b**) loss angle curves of PLA, PBAT, and their blends.

The effect of MA–PBAT on the viscoelastic properties of PLA blends was investigated through DMA analysis, with the temperature-dependent curves of storage modulus and loss angle shown in Figure 10. Compared to pure PLA, the composite materials exhibited relatively lower storage moduli, attributable to the low-storage-modulus characteristics of the PBAT phase, indicating reduced material rigidity. Furthermore, as the MA concentration increased, the initial modulus demonstrated a gradual upward trend accompanied by partial restoration of rigidity, which aligns with the mechanical test results. Generally, the peak temperature of the loss tangent corresponds to the glass transition temperature of the material [38]. The glass transition temperature progressively decreased with increasing MA concentration, suggesting enhanced compatibility between the material components.

### 3.5. Rheology Properties

**Figure 11:**
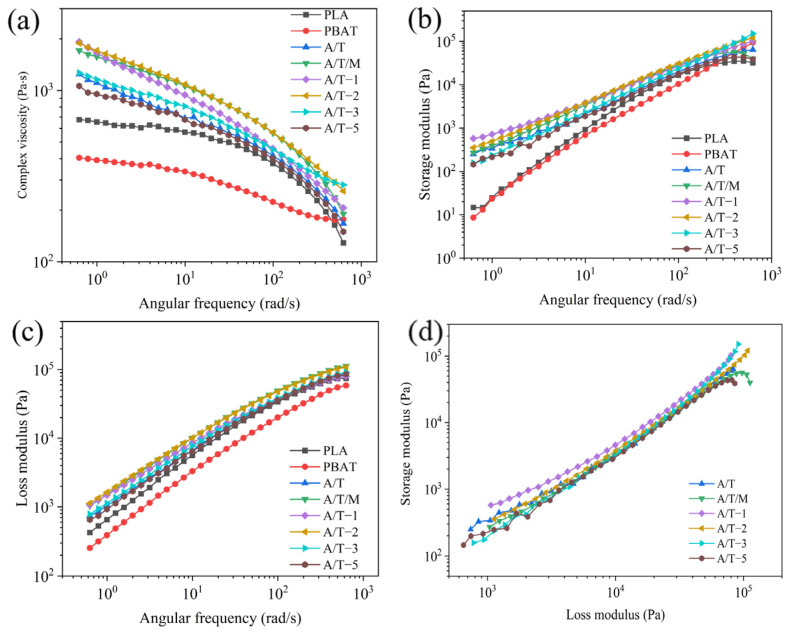
Rheological properties of composites: (**a**) complex viscosity, (**b**) storage modulus, (**c**) loss modulus, and (**d**) Han curve.

To better elucidate the evolution of the morphology of the blend, the interrelationship among the fluidity of pure PLA, PBAT, and the blend was examined under dynamic circumstances, as illustrated in Figure 11. All samples manifested varying degrees of shear thinning (Figure 11a). Compared with the pure samples without the addition of MA, the majority of modified materials exhibit higher viscosity. This can be attributed to the fact that the grafted MA enhances the entanglement capability between the two phases. The increase in entanglement capability reduces the fluidity of the system, manifested as an increase in viscosity [39]. Nevertheless, as the content of MA kept increasing, the viscosity of the samples reached a peak and then gradually declined. This is because at low concentrations, the MA grafted onto PBAT augmented the reaction sites with PLA, facilitating a tighter combination of the two phases. However, an excessive amount of MA would cause cross-linking among PBAT chain segments, forming a PBAT–MA–PBAT structure, thereby hindering the connection with PLA. At this stage, MA–PBAT would disperse among PLA chain segments, functioning as a plasticizer and reducing the complex viscosity of the composite. The complex viscosity of the mixture obtained by blending PBAT–MA treated with MA and PLA is higher than that of the A/T mixture and the A/T/M mixture with direct addition of MA. This indicates that PBAT–MA has superior reactivity, thereby generating more prominent chain extension effects and consequently enhancing the performance of the composite material more effectively.

As depicted in Figure 11b,c, as the angular frequency accelerates, the modulus of the blend system shows an upward trend. The variation range of the loss moduli of pure PLA, A/T, A/T/M, and A/T-5 is greater than that of the storage modulus, dominated by viscous behavior. The variation range of the loss moduli of other component materials is smaller than that of the storage modulus, dominated by elastic behavior. The addition of both PBAT and PBAT–MA enhances the modulus of PLA material. This is because a crosslinked structure is formed after blending, making the entanglement of molecular chain segments more compact and therefore consuming more energy during the flow. In contrast, the enhancement of the two moduli by PBAT–MA is more significant. However, consistent with the complex viscosity, an overly high concentration of MA will reduce the flexibility of the PBAT chain segments, making it more difficult for them to be compatible with PLA, thereby reducing these two moduli.

The widely employed Han curve can comparatively and intuitively illustrate the compatibility of polymer blends in two-phase systems. As depicted in Figure 11d, the Han curves of the A/T/M and A/T-5 blends demonstrate significant deviations in the high-frequency region, suggesting relatively poor compatibility at this point. Curves for MA content of 1 wt%, 3 wt%, and 5 wt% show excellent linearity within the frequency range, indicating that the addition of MA enhances the interfacial adhesion between molecules, enabling better entanglement among molecules and thereby improving the compatibility.

### 3.6. Toughening Mechanism

**Figure 12:**
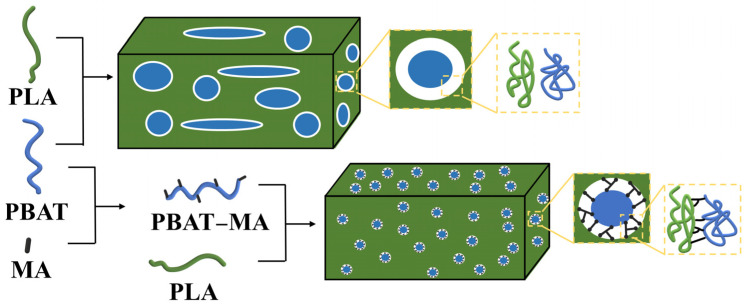
Mechanism of reinforcing toughness between PLA and PBAT.

Based on the experimental findings presented, the toughening mechanism of PBAT–MA for polylactide is illustrated in Figure 12. Following its reaction with MA, PBAT generates multiple reactive sites within its chain segments, thereby enhancing the likelihood of cross-linking between polylactide and PBAT. Given that MA reacts prior to PBAT, during the melt-blending process of PBAT–MA and PLA, a greater quantity of PLA–MA–PBAT copolymers is formed. This effectively precludes the formation of PLA–MA–PLA copolymers, resulting in a marked reduction in low-toughness structures and facilitating enhanced compatibility between PLA and PBAT, ultimately leading to improved toughness of the final product.

## 4. Conclusions

In this paper, PBAT–MA was obtained by modifying PBAT with MA as a modifier, and then a PLA–PBAT blend was obtained by melt-blending with PLA. The modified PLA–PBAT blend was compared with the unmodified A/T blend and the A/T/M blend obtained by directly adding MA during the blending of PLA and PBAT, with a focus on the influence on mechanical properties. According to the results of nuclear magnetic resonance and infrared spectroscopy, it was observed that MA was successfully grafted onto PBAT, increasing the reaction sites between PLA and PBAT and enhancing the compatibility. When the addition of MA reached 2 wt%, the toughness of the material was improved, with an elongation at break of 358.1% and an impact strength of 333.9 kJ/m^2^, which were 24.6 times and 18.7 times that of pure PLA, respectively, and 4.7 times and 6.6 times that of the A/T blend obtained by directly adding MA. However, the strength decreased to 34.0 MPa, demonstrating that this method effectively improved the toughness. Additionally, the thermal stability of the polymer was also enhanced. The rheological results indicate that the MA-modified PBAT can provide higher system viscosity and moduli for the A/T blend, and the Han curve suggests that the compatibility has been improved. The structure of MA grafted onto PBAT leads to the formation of more PLA–MA–PBAT structures and reduces the occurrence of PLA–MA–PLA structures, enabling better stress transfer between PLA and PBAT, thereby achieving better toughness.

## Figures and Tables

**Table 1:** Formulation of the samples investigated.

Sample	PLA (wt%) ^a^	PBAT (wt%) ^a^	PBAT–MA (wt%) ^a^	MA (wt%) ^b^	BPO (wt%) ^b^
A/T	70	30	0	0	0
A/T/M	70	30	0	2	1
A/T—1	70	0	30	1	1
A/T—2	70	0	30	2	1
A/T—3	70	0	30	3	1
A/T—5	70	0	30	5	1

^a^ Based on the weight percentage of the whole quantity, ^b^ based on the weight percentage of PBAT or PBAT–MA.

**Table 2:** Mechanical property characterization of composites.

Sample	Tensile Strength (MPa)	Impact Strength (kJ/m^2^)	Elongation at break (%)	Young’s Modulus (MPa)
PLA	60.6 ± 1.1	17.8 ± 2.4	14.6 ± 2.3	850.9 ± 81.3
A/T	40.3 ± 2.3	36.4 ± 4.5	79.5 ± 7.9	638.8 ± 33.4
A/T/M	37.0 ± 1.2	50.4 ± 4.9	76.0 ± 3.4	498.2 ± 28.7
A/T−1	31.9 ± 1.6	96.9 ± 9.6	96.1 ± 12.7	401.1 ± 27.8
A/T−2	34.0 ± 1.2	333.9 ± 21.2	358.1 ± 18.0	472.2 ± 38.9
A/T−3	36.1 ± 1.1	67.5 ± 4.1	126.1 ± 4.6	412.2 ± 32.2
A/T−5	42.2 ± 2.2	46.3 ± 4.6	69.9 ± 15.8	511.8 ± 44.8

**Table 3:** Thermal behavior data of samples.

Sample	T_g1_ (°C)	T_g2_ (°C)	ΔT_g_ (°C)	T_cc_ (°C)	T_m1_ (°C)	T_m2_ (°C)	ΔH_m_ (J/g)	X_c_ (%)
PLA	—	60.2	—	124.2	161.8	—	0.7	0.8
PBAT	−30.1	—	—	—	119.9	—	14.9	13.1
A/T	−32.8	60.6	93.4	103.8	—	164.1	3.2	3.2
A/T/M	−34.1	60.3	94.4	108.2	157.9	164.5	2.2	2.3
A/T−1	−31.2	60.3	91.5	106.9	—	165.6	2.2	2.2
A/T−2	−30.8	59.3	90.1	108.1	158.6	164.7	4.3	4.3
A/T−3	−31.6	59.5	91.1	108.1	158.8	164.9	3.9	3.9
A/T−5	−33.1	60.5	93.6	107.7	158.7	164.9	3.2	3.2

— This indicates that there are no corresponding data.

**Table 4:** Thermal properties of samples.

Formulation	T_5%_ (°C)	T_50%_ (°C)	T_final_ (°C)	T_max1_ (°C)	T_max2_ (°C)
PLA	311.8	348.3	369.4	352.8	—
PBAT	344.7	386.3	494.1	—	388.8
A/T	310.1	348.6	475.5	344.7	379.3
A/T/M	302.6	347.9	471.1	344.7	384.9
A/T-1	317.1	351.2	487.4	347.4	383.7
A/T-2	324.8	354.2	493.0	350.0	389.7
A/T-3	310.0	346.2	476.3	341.3	379.6
A/T-5	306.1	341.3	470.8	336.4	379.0

— This indicates that there are no corresponding data.

## Data Availability

The original contributions presented in the study are included in the article, further in-quiries can be directed to the corresponding author.
